# Low-profile 2.7 mm VA-LCP plates vs. 3.5 mm LCP plates for midshaft clavicle osteosynthesis: a retrospective comparison

**DOI:** 10.1007/s00068-026-03110-x

**Published:** 2026-03-04

**Authors:** D. Brun, Y. Lecoultre, B. J. M. van de Wall, F. Pretz, S. Geuss, R. Babst, B. C. Link, F. J. P. Beeres

**Affiliations:** 1 Faculty of Health Sciences and Medicine, University of Lucerne, Lucerne, Switzerland; 2 Department of Orthopedic and Trauma Surgery, LUKS Lucerne, Lucerne, Switzerland

**Keywords:** Clavicle fractures, Single plate osteosynthesis, Irritation, Plate removal, Plate design

## Abstract

**Purpose:**

Implant related irritation and subsequent removal are one of the main disadvantages of clavicle plate osteosynthesis. 2.7 mm VA-LCP clavicle plates with a lower profile were introduced in recent years, with the aim to improve fit and reduce plate prominence.

This study aims to investigate whether this leads to a lower implant removal rate compared to the older 3.5 mm LCP clavicle plates.

**Methods:**

All patients that underwent single-plate osteosynthesis for midshaft clavicle fractures between 2019 and 2024 in a Swiss level I trauma-center were included. Patients were categorized into two treatment groups: DePuy Synthes 3.5 mm LCP clavicle plates or DePuy Synthes 2.7 mm VA-LCP clavicle plates. Treatment groups were compared regarding healing, complications, and removal rate.

**Results:**

101 patients were included, 54 with a 2.7 mm VA-LCP plate, and 47 with a 3.5 mm LCP plate. There were no significant differences in implant removal due to hardware irritation (44% versus 49%), overall reintervention rate (46.3% versus 53.2%, *p* = 0.49), infection (0% versus 4.3% *p* = 0.21), and refractures (1.9% versus 6.4%, *p*=0.34). All fractures healed in both groups.

**Conclusion:**

Newer 2.7 mm VA-LCP clavicle single plating shows similar results in terms of complication and healing rates compared to older 3.5 mm LCP plating. Despite the design aiming for reduced plate-prominence, it does not demonstrate a significant reduction in removal rates in this study. Further studies are needed to ascertain the generalizability of results.

## Introduction

Clavicle fractures are among the most common injuries to the shoulder girdle and mostly affect young, active patients. Around two thirds of these fractures occur at the midshaft [1, 2]. Surgical fixation has proven to expedite healing and reduce non-unions compared to nonoperative management, allowing for earlier return to work and sports. Therefore, it has become the standard treatment for displaced fractures in many hospitals, with the most common fixation technique being plate osteosynthesis [3–6].

However, plate prominence under the thin soft-tissue layer covering the clavicle often leads to irritation and discomfort, and subsequently to plate removal in up to 60% [7, 8]. This additional surgery comes with the risk of refracture of up to 7% [9, 10].

Often irritation is caused by poor geometric fit and plate prominence, suggesting improving implants and their positioning could lead to a lower removal rate [11]. One attempt to reduce irritation was anterior plate positioning, which proved to have no influence on removal rates as well as functional improvement and pain levels [8].

In recent years, new plate designs with a lower profile and more accurate plate-to-bone fit were introduced, one being the 2.7 mm VA-LCP Clavicle System by DePuy Synthes. These implants use variable-angle locking screws with a diameter of 2.7 mm and a smaller plate-screw interface than the older 3.5 mm LCP clavicle plates which use 3.5 mm screws. Therefore, the 2.7 mm VA-LCP plates are thinner and have a lower profile than the 3.5 mm LCP plates. These implants have been used in clinical practice since 2021 and shown to be reliable in terms of biomechanical stability [12]. However, until now there have been no studies on their clinical performance. Therefore, a retrospective comparative study was performed evaluating removal rates, complications and healing of both the older 3.5 mm LCP clavicle plate system versus the newer 2.7 mm VA-LCP clavicle plates.

## Materials and methods

### Study design and population

**Fig. 1:**
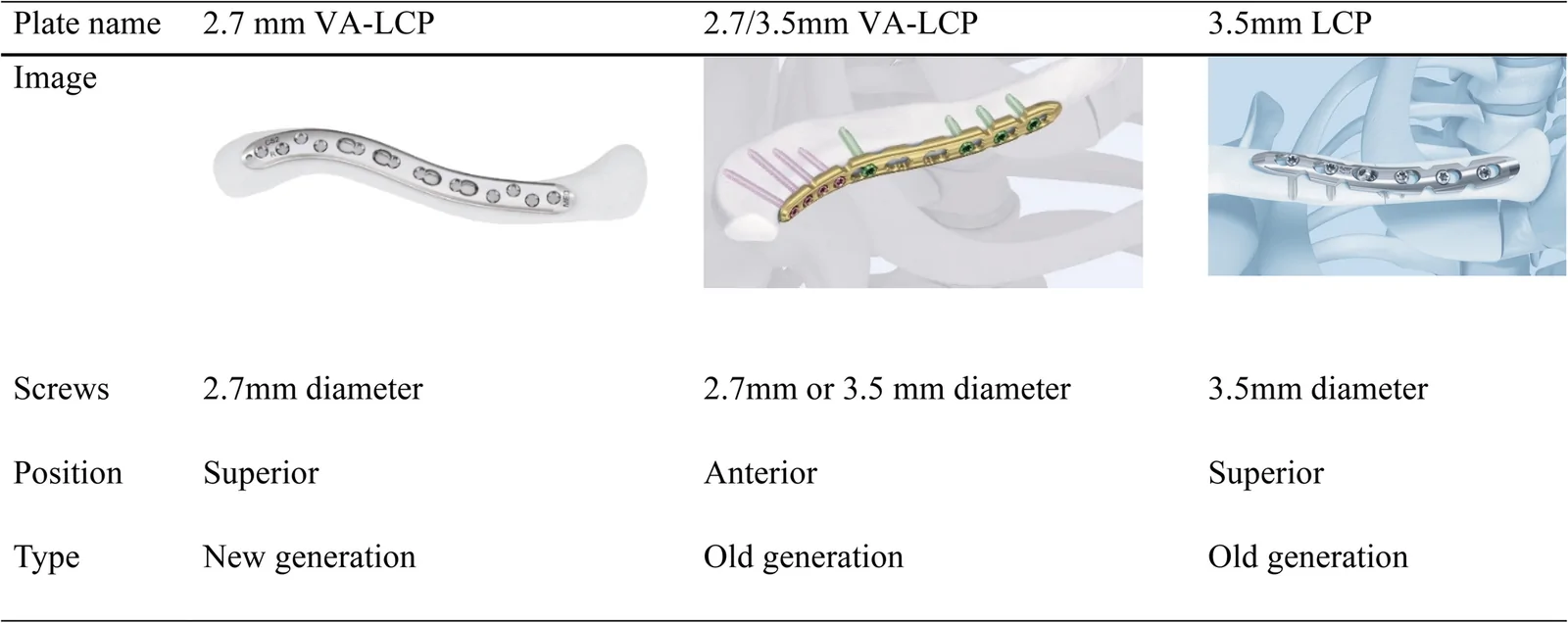
Plate characteristics, product images by courtesy of DePuy synthes

All adult patients (> 16 years) surgically treated for a clavicle fracture at the study hospital between October 2019 and January 2024 were screened. Patients treated with a single plate for a midshaft fracture (AO 12.2 A, B, or C) and a minimum follow-up of 12 months were included. The implants that were included were either the newer VA-LCP 2.7 mm clavicle plate or an older 3.5 mm LCP superior and the 2.7/3.5 mm VA-LCP anterior clavicle plate. An illustration of these implants is shown in Fig. 1. A previous study has shown that plate position does not have an effect on implant removal or irritation [8], therefore the 3.5 mm LCP superior and the 2.7/3.5 mm VA-LCP anterior clavicle plate were put into the same study group for analysis. This group will be mentioned as 3.5 mm LCP clavicle plate. Additionally, a subgroup analysis comparing only superior implants was performed. Exclusion criteria encompassed refractures, open and pathologic fractures. The underlying protocol was approved by the Northwest and Central Swiss Ethics Committee (nr. 2020 − 00625). A standardized approach for retrospective observational studies at the study center was applied [13].

### Data extraction

Hospital records were accessed on March 1, 2025, and data were extracted to a pseudonymized database. Baseline characteristics included age, gender, body mass index (BMI), American Society of Anesthesiologists (ASA) score, high/low energy trauma (according to the definition of the Advanced Trauma Life Support, ATLS), Injury Severity score (ISS), concomitant injuries and fracture type according to OTA/AO. One reviewer classified all fractures based on the X-rays obtained at presentation. These findings were controlled against the radiologist’s report. If there was disagreement, a third researcher was consulted.

Surgical data was extracted from the surgical reports and included time interval between presentation and surgery, plate positioning (anterior, superior) and type of implant, use of lag screws, operation duration, complications, length of hospital stay, and experience level of the surgeon.

### Surgical indication, procedure and aftercare

The indication for a surgical approach was based on fracture displacement of more than one shaft-width or shortening by >2 cm as well as patient characteristics such as age and activity level.

All procedures were performed using a standardized open approach. An incision was made in the skinlines over the fracturezone in the clavicle, followed by dissection through the subcutaneous tissue and visualization of the fracture. The supraclavicular nerves were not routinely spared. Pectoral muscles were sparingly detached if needed. After fracture reduction, the plate was positioned either superiorly or anteriorly, depending on implant type and surgeon preference. Wound closure was performed in three layers, ensuring adequate soft tissue coverage: The platysma and subcutis were closed with absorbable braided sutures (size 3 − 0) and skin was closed using a non-absorbable, monofilament synthetic suture (size 4 − 0) in Allgöwer technique.

Before June 2021, only the 3.5 mm LCP plates were available. Afterwards, they were replaced by the 2.7 mm VA-LCP plates.

Older 3.5 mm LCP clavicle shaft plates were implanted either in a superior or anterior position. For the new 2.7 mm VA-LCP clavicle plate design, only a superior position is possible. Lag screws or reposition plates were used where needed. The plates were fixed using cortical and/or locking screws.

Aftercare was the same for both implants. For the first 2 weeks shoulder abduction/flexion was limited to 90 degrees. Patients were instructed to non-weight bear for 6 weeks. Afterwards, patients were allowed to move freely and progressively bear weight. All patients were routinely planned for follow-up visits 6 weeks, 3 months and one year after surgery with radiographies of the operated clavicle. Implant removal was discussed routinely at the 12 months visit and only offered to patients suffering from implant irritation. Implant removal was planned between 12 and 18 months after the initial surgery.

### Primary outcome

The primary outcome was implant removal due to irritation. To measure this outcome the hospital records of the 12-month visit (or later) were screened for the occurrence of irritation and subsequent planning of a removal.

### Secondary outcomes

Secondary outcomes included all adverse events such as implant failure, infections and non-union. Implant failure was defined as any sign of screw or plate loosening, breakage or bending. Fracture-related infections were diagnosed using the definition of Metsemakers et al. [14]. Non-union was defined as the absence of bridging callus or fading of fracture lines in three out of four cortices in the anteroposterior and lateral X-ray views. Additionally, operation duration was analyzed.

### Statistical analysis

Statistical analysis was performed using SPSS (IBM SPSS Statistics 29.0.2.0). The median with interquartile range (IQR) was calculated for non-normally and mean with corresponding standard deviation (SD) for normally distributed data. Percentages were calculated for all categorical variables. Groups were compared using either chi-square, Fisher’s exact, Students T-test (normal data), or Mann-Whitney U (non-normal data). A *p*-value threshold of 0.05 was considered significant.

## Results

### Patient selection and characteristics

**Fig. 2:**
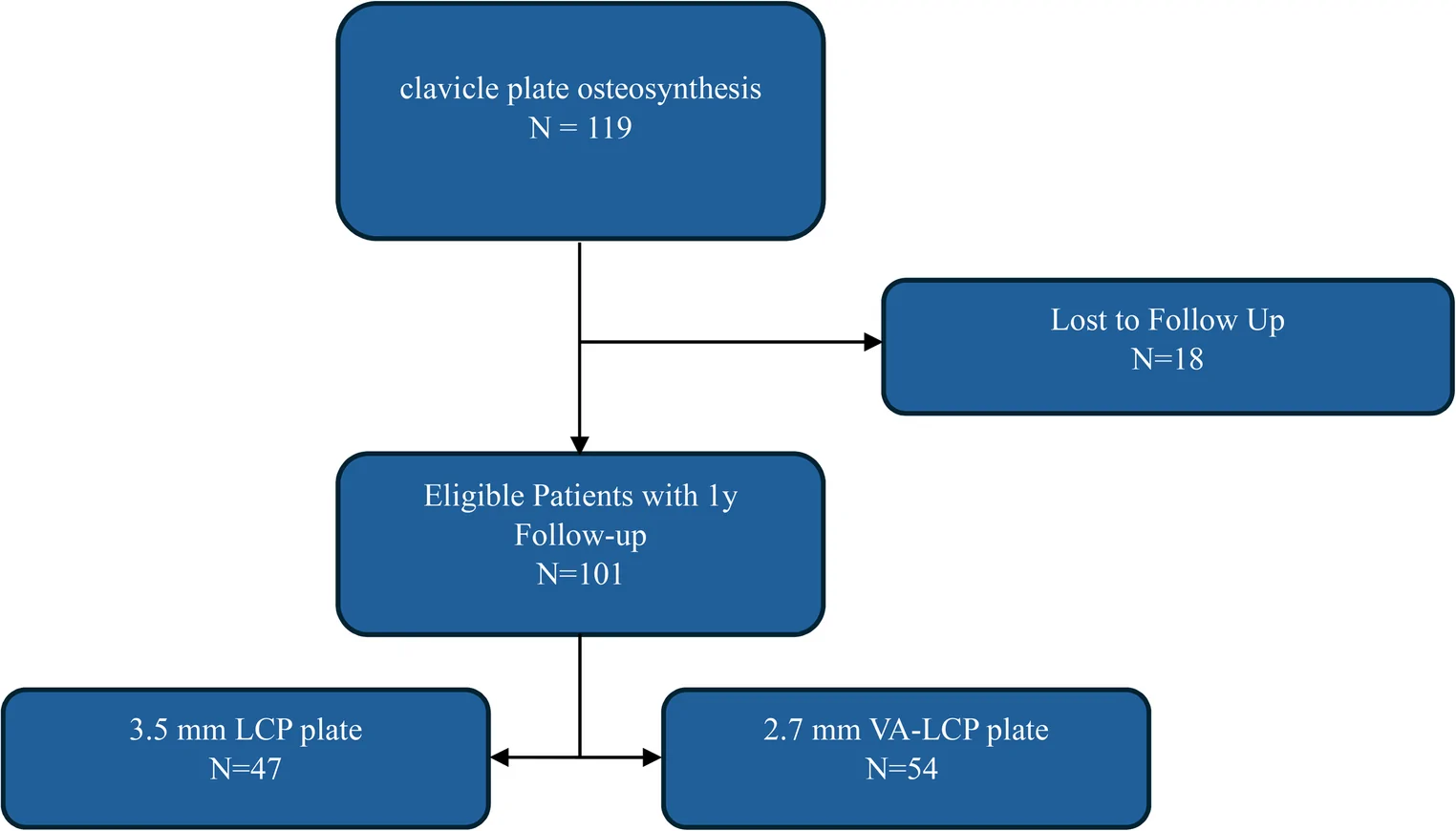
Inclusion process

In the study period, a total of 119 patients underwent plate osteosynthesis for a clavicle fracture using either 3.5 mm LCP or a 2.7 mm VA-LCP clavicle plate. Of these, 101 met the inclusion criteria: 54 patients with a 2.7 mm VA-LCP plate and 47 patients with a 3.5 mm LCP plate (Fig. 2). Baseline characteristics did not differ between the groups. A detailed description is shown in Table 1.

**Table 1:** Baseline characteristics

	3.5 mm LCP (*N* = 47)	2.7 mm VA-LCP(*N* = 54)	*p*-value
Age (y) (mean ± SD)	44 ± 16	47 ± 17	*0.49*
Gender (female)	5 (10.6%)	11 (20.4%)	0.18
BMI (kg/m2) (mean ± SD)	25.2 ± 3.6	25.0 ± 37	0.78
Smoking	19 (36.2%)	16 (25.9%)	0.27
Diabetes	2 (4.3%)	2 (3.7%)	0.89
ASA classification			0.86
I	13 (27.7%)	15 (28.3%)	0.81
II	26 (55.3%)	29 (54.7%)	
III	8 (17.0%	8 (15.1%)	
IV	0	1 (1.9%)	
V	0	0	
High energy trauma	9 (19.1%)	15 (27.8%)	0.31
Polytrauma	5 (10.6%)	9 (16.7%)	0.38
Other injuries in different body part	24 (51.1%)	31 (57.4%)	0.52
Injury on ipsilateral extremity	18 (38.3%)	25 (46.3%)	0.42
Shortening	17 (36.2%)	17 (31.5%)	0.62
Dislocation	37 (78.7%)	48 (88.9%)	0.16
AO fracture type			0.65
A (simple)	9 (19.1%)	11 (20.4%)	
B (wedge)	5 (10.6%)	9 (16.7%)	
C (multi-fragmentary)	33 (70.2%)	34 (63.0%)	

### Surgical data

For the 3.5 mm LCP plates, the surgeons selected a superior position in eleven cases (23.4%). The newer 2.7 mm VA-LCP plate can only be implanted superiorly by default as previously stated.

Mean operation duration was significantly lower in the 2.7 mm VA-LCP plating group (118 min for the 3.5 mm LCP group vs. 101 min for the 2.7 mm VA-LCP group, *p* = 0.006).

There was one iatrogenic pneumothorax in the 3.5 mm LCP group that was managed conservatively. No other surgery related perioperative complications were recorded.

Fifty (49.5%) surgeries were performed by surgical residents. There was no significant difference in experience between both implant groups. All surgical data can be found in Table 2.

**Table 2:** Surgical data

	3.5 mm LCP (*N* = 47)	2.7 mm VA-LCP(*N* = 54)	*p*-value
Implant position (superior)	11 (23.4%)	54 (100%)	
Implant position (anterior)	36 (76.6%)	0 (0.0%)	
Reposition plate	6 (12.8%)	0	
Lag screw	33 (70.2%)	38 (70.4%)	0.99
Operation time (min)	118 ± 33 (*N* = 45)	101 ± 24 (*N* = 44)	0.006
Time to surgery (d) (median, IQR)	2 (3)	3 (5)	0.13
Stay after surgery (d) (median, IQR)	2 (1)	2 (3)	0.94
Experience of the Surgeon			0.51
Resident in training	25 (53.2%)	25 (46.3%)	
Fellow/Consultant	14 (29.8%)	22 (40.7%)	
Senior Consultant/Chief Surgeon	8 (17.0%)	7 (13.0%)	

### Primary outcome

There was a total of 25 (53.2%) reinterventions in the 3.5 mm LCP group compared to 25 (46.3%) in the 2.7 mm VA-LCP group, showing no significant differences. The main reason for reintervention was hardware irritation accounting for 23 operations (48.9%) in the 3.5 mm LCP and 24 (44.4%) in the 2.7 mm VA-LCP group (Table 3).

**Table 3:** Primary and secondary outcomes

	3.5 mm LCP (*N* = 47)	2.7 mm VA-LCP(*N* = 54)	*p*-value
Reintervention	25 (53.2%)	25 (46.3%)	0.49
Reintervention reason			0.32
Hardware irritation	23 (48.9%)	24 (44.4%)	
Infection	2 (4.3%)	0 (0%)	
Implant failure	0 (0%)	1 (1.9%)	
Refracture after removal	3 (6.4%)	1 (1.9%)	0.34
Subgroup analysis	3.5 mm LCP superior (*N* = 11)	2.7 mm VA-LCP (*N* = 54)	
Reintervention	4 (36.4%)	25 (46.3%)	0.55
Operation time (min)	105 ± 30 (*N* = 10)	101 ± 24 (*N* = 44)	0.55

### Secondary outcomes

One implant failure (loss of reduction due to screw loosening) was reported in the 2.7 mm VA-LCP group occurring after heavy lifting 4 months after surgery. There were two cases of fracture-related infections in the 3.5 mm LCP group, with one requiring revision surgery with implant-replacement and antibiotic therapy.

There were no non- /malunions in either of the groups. Four refractures occurred after material removal, three of which were in the 3.5 mm LCP group. The refractures occurred 2, 6 and 15 weeks after implant removal. One refracture initially was asymptomatic and the patient presented himself with a symptomatic pseudoarthrosis 3 years after removal. All of them were treated with re-osteosynthesis and eventually healed.

### Subgroup analysis for superior plates only

To address potential confounding by plate position, a subgroup analysis of plates implanted in a superior position was performed. In the 3.5 mm LCP group, a total of eleven patients (23.4%) received a superior plate. Data of these patients was compared to all patients that received a 2.7 mm VA-LCP plate. There were no significant differences in clinical outcomes (36.4% vs. 46.3% reinterventions, *p* = 0.55). There was no significant difference in operative time when only comparing superior plating (mean 105 vs. 101 min, *p* = 0.55).

## Discussion

This retrospective analysis comparing new 2.7 mm VA-LCP to old 3.5 mm LCP implants did not show any significant differences in implant removal rates caused by hardware irritation (44% (2.7 mm) vs. 49% (3.5 mm) or overall re-interventions (46% vs. 53%, *p* = 0.49). Non-union or implant failure were equally rare in both groups. There was a significantly lower operation duration in the 2.7 mm VA-LCP plate group (101 min compared to 118 min, *p* = 0.006).

### Interpretation of results

To date, this is the first study analyzing the clinical performance of new 2.7 mm VA-LCP clavicle plates and comparing it to older 3.5 mm LCP plates.

Clavicle osteosynthesis is plagued by high irritation rates [7, 8]. This has prompted the development of next-generation implant designs, such as 2.7 mm VA-LCP plates, aimed at minimizing soft tissue irritation. However, when comparing the implant removal and irritation rates associated with the 2.7 mm VA-LCP system to those of 3.5 mm LCP plates, no statistically significant benefit could be demonstrated.

A possible explanation is that the designation “2.7 mm” refers to the diameter of the screws used rather than the plate size itself. Although the 2.7 mm VA-LCP system features a reduced plate thickness (2.6 mm versus 3.0 mm), the plate width remains nearly unchanged (10.1 mm versus 10.3 mm) to preserve biomechanical integrity [15]. Given the limited soft tissue envelope overlying the clavicle, this slight reduction in plate dimensions does not seem sufficient to reduce irritation.

Interestingly, use of the 2.7 mm VA-LCP plates was associated with a statistically significant reduction in operative time (118 vs. 103 min). However, this difference appears to be driven primarily by implant positioning rather than implant design. When the analysis was restricted to cases with superior plate positioning, the operative time difference between the two systems decreased to only 4 min. This subgroup analysis, however, was limited by the small number of cases in the superior 3.5 mm LCP group, resulting in limited statistical power to detect a difference.

Notably, fixation with 2.7 mm VA-LCP plates requires four screws on each side of the fracture, according to the implant manual, compared with three screws for 3.5 mm LCP plates. Thus, despite the greater number of fixation steps, operative time remained comparable in the subgroup analysis, suggesting a potential procedural advantage of the newer plate system. One plausible explanation is the improved precontouring of the 2.7 mm VA-LCP plates, which may reduce the time required for plate bending and positioning, thereby compensating for the additional time needed to place more screws. Nevertheless, neither the clinical benefit nor the biomechanical necessity of the increased screw number mandated by the 2.7 mm VA-LCP system has been reported. This raises the question of whether four screws are truly required, as reducing the screw count for the 2.7 mm plates could render differences in operative time both clinically and statistically significant.

Interestingly, refractures following plate removal were observed predominantly in the 3.5 mm LCP group. Although the rarity of this complication precludes formal statistical analysis, the trend raises potentially meaningful consideration. 3.5 mm LCP plates typically require three 3.5 mm screws, whereas the newer 2.7 mm VA-LCP plates are secured with four smaller screws. It is plausible that the smaller diameter of the screw holes left after removal of the 2.7 mm VA-LCP plate may reduce the structural weakening of the clavicle, thereby decreasing the risk of refracture. While this observation suggests a possible biomechanical advantage of the newer plate design, larger studies are necessary to confirm its clinical significance.

The 2.7 mm VA-LCP plates offers a viable alternative in the treatment of clavicle osteosynthesis and may offer small benefits in terms of operative time and reduced refracture rates, but it does not show a significant reduction in implant irritation in this study. This highlights the ongoing need for innovation in clavicle osteosynthesis. To further reduce implant irritation, implant thickness and width could be reduced even further. This however may come with an increased risk of implant failure due to the decreased biomechanical stability of thinner/smaller plates. A promising approach is low-profile double-plating using two mini-fragment plates, which offer an the lower plate prominence, theoretically reducing implant irritation while retaining biomechanical stability [16]. Being a fairly new technique, it remains to be seen how they compare to the 2.7 mm VA-LCP plates.

### Limitations

A potential confounder in this study is plate positioning, as many of the older 3.5 mm LCP plates were implanted in an anterior position. Although a recently published large-scale multicenter analysis reported no statistically significant differences in implant removal rates (59% vs. 57%, *p* = 0.88), time to healing (48 vs. 52 weeks, *p* = 0.64), or overall complication rates between anterior and superior plating (8), confounding introduced by implant position cannot be entirely ruled out. Notably, subgroup analysis restricted to superior plating also failed to reveal significant differences in removal or irritation rates. However, the limited number of 3.5 mm LCP plates placed superiorly reduced statistical power.

Also, this study was designed as a single center retrospective analysis. Even though no differences in baseline characteristics between treatment groups were detected, confounding by unmeasured characteristics is not excluded. Lastly, this was a single center study. It remains to be seen whether similar results will be found in other hospitals and, with it, the generalizability of results of the present study.

## Conclusion

Patients in the present study treated with the newer 2.7 mm VA-LCP clavicle plates showed similar healing and complication rates as patients treated with the older 3.5 mm LCP plates. Despite the reduced plate-prominence design of the new 2.7 mm plate, no reduction in implant removal rate due to irritation could be demonstrated. As this is the first study to investigate the new plate, it remains to be seen whether similar results will be found in future studies.

## Data Availability

Raw data for this dataset is not publicly available to preserve individuals’ privacy under the European General Data Protection Regulation.

## References

[1] Postacchini F, Gumina S, De Santis P, Albo F. Epidemiology of clavicle fractures. (in eng). J Shoulder Elb Surg. 2002;11(5):452–6. 10.1067/mse.2002.126613. 12378163

[2] Kihlström C, Möller M, Lönn K, Wolf O. Clavicle fractures: epidemiology, classification and treatment of 2 422 fractures in the Swedish fracture Register; an observational study. BMC Musculoskelet Disord. 2017;18(1). 10.1186/s12891-017-1444-1. PMC5312264 28202071

[3] Wiesel B, Nagda S, Mehta S, Churchill R. Management of midshaft clavicle fractures in Adults. (in eng). J Am Acad Orthop Surg. Nov 15 2018;26(22):e468–76. 10.5435/JAAOS-D-17-00442. 30180095

[4] Von C, Rüden, et al. Evidence on treatment of clavicle fractures. Injury. 2023;54(110818). 10.1016/j.injury.2023.05.049. 37217399

[5] Woltz S, et al. Plate fixation compared with nonoperative treatment for displaced midshaft clavicular fractures: A multicenter randomized controlled Trial (in eng). J Bone Joint Surg Am. 2017;99(2):106–12. 10.2106/JBJS.15.01394. 28099300

[6] Martin JR, et al. Comparative effectiveness of treatment options for displaced midshaft clavicle fractures. Bone Joint Open. 2021;2(8):646–54. 10.1302/2633-1462.28.bjo-2021-0112.r1. PMC8384438 34402306

[7] Hulsmans MHJ, et al. High irritation and removal rates after plate or nail fixation in patients with displaced midshaft clavicle fractures. Clin Orthop Relat Res. 2017;475(2):532–9. 10.1007/s11999-016-5113-8. PMC5213945 27830484

[8] Buenter IR, et al. Does plate position influence the outcome in midshaft clavicular fractures? A multicenter analysis. Eur J Trauma Emerg Surg. 2024;50(3):1023–31. 10.1007/s00068-023-02400-y. PMC11249707 38231234

[9] Zhu Y, Hu J, Zhan T, Zhu K, Zhang C. Refracture after plate removal of midshaft clavicle fractures after bone union—incidence, risk factors, management and outcomes. BMC Musculoskelet Disord. 2023;24(1). 10.1186/s12891-023-06391-0. PMC10114427 37076821

[10] Tsai S-W, et al. Risk factors for refracture after plate removal for midshaft clavicle fracture after bone union. J Orthop Surg Res. 2019;14(1). 10.1186/s13018-019-1516-z. PMC6925412 31864405

[11] Vancleef S, Wesseling M, Duflou JR, Nijs S, Jonkers I. Vander Sloten. Thin patient-specific clavicle fracture fixation plates can mechanically outperform commercial plates: an in Silico approach. J Orthop Res. 2022;40(7):1695–706. 10.1002/jor.25178. 34668224

[12] Alzahrani MM, Cota A, Alkhelaifi K, Harvey EJ Mechanical evaluation of 2.7- versus 3.5-mm plating constructs for midshaft clavicle Fractures (in eng). J Am Acad Orthop Surg. 2021;29(9):e440–6. 10.5435/JAAOS-D-19-00495. 32701682

[13] Von Elm E, Altman DG, Egger M, Pocock SJ, Gøtzsche PC. Vandenbroucke. The strengthening the reporting of observational studies in epidemiology (STROBE) statement: guidelines for reporting observational studies. PLoS Med. 2007;4(10):e296. 10.1371/journal.pmed.0040296. PMC2020495 17941714

[14] Metsemakers W, et al. Fracture-related infection: A consensus on definition from an international expert group. Injury. 2018;49(3):505–10. 10.1016/j.injury.2017.08.040. 28867644

[15] Synthes DP. 2.7 MM VA LCP™ CLAVICLE System. https://www.jnjmedtech.com/en-US/product/variable-angle-lcp-clavicle-system. Accessed 10/07/2025.

[16] Lecoultre Y, Van De Wall B, Link B-C, Kik C, Babst R, Beeres F. Low-profile double plating versus conventional single plating in midshaft clavicle fractures: A retrospective study and initial experience with this novel technique. PLoS ONE. 2025;20(1):e0318004. 10.1371/journal.pone.0318004. PMC11759345 39854480

